# Public opinion on alcohol consumption and intoxication at Swedish professional football events

**DOI:** 10.1186/s13011-017-0103-8

**Published:** 2017-05-08

**Authors:** Charlotte Skoglund, Natalie Durbeej, Tobias H. Elgán, Johanna Gripenberg

**Affiliations:** STAD, Centre for Psychiatry Research, Department of Clinical Neuroscience, Karolinska Institutet, & Stockholm Health Care Services, Stockholm County Council, Norra Stationsgatan 69, SE-113 64 Stockholm, Sweden

**Keywords:** Public opinion Alcohol intoxication Football Sporting events AUDIT-C

## Abstract

**Background:**

Alcohol-related problems at professional sporting events are of increasing concern and alarming reports are often reported in international media. Although alcohol consumption increases the risk for interpersonal violence, it is viewed as a focal element of large football events. Sweden has a long tradition of high public support for strict alcohol-control policies. However, little is known about public opinions on alcohol intoxication and the support for interventions to decrease intoxication at football events. The current study explored the public opinion towards alcohol use, intoxication and alcohol policies at professional football matches in Sweden.

**Methods:**

A cross-sectional design was utilized and a random general population sample of 3503 adult Swedish residents was asked to participate in a web survey during 2016 (response rate 68%).

**Results:**

In total, 26% of the respondents supported alcohol sales at football events. Over 90% reported that obviously intoxicated spectators should be denied entrance or evicted from arenas. The support for regulations limiting alcohol availability varied with background factors such as gender, alcohol use and frequency of football event attendance.

**Conclusions:**

There is a strong public consensus for strategies and policies to reduce alcohol sales and intoxication levels at football matches. This public support has implications for our preventive efforts and will facilitate the implementation of strategies and policy changes.

## Highlights

- Alcohol-related disturbances at or near stadiums hosting professional football matches are common.
- There is a strong consensus for reducing intoxication levels at football matches.
- Support for regulations varied by gender and individual hazardous alcohol use.

## Background

In recent years, there has been an ongoing discussion in both international [1, 2] and Swedish media [3, 4] about the increasing problems of interpersonal violence and disturbances associated with professional sporting events. There is mounting evidence that alcohol consumption contributes considerably to a range of adverse and undesirable outcomes such as violence at professional sporting events [5–9]. However, alcohol use among spectators is often viewed as a focal element of sporting events. Alcohol consumption is associated with various aspects of impulsivity, aggression and difficulties in regulating emotions. In particular, high-risk alcohol consumption, i.e., heavy episodic drinking and intoxication, increases the risk for violence [10–12]. In Sweden, the situation of intoxication and violence, in particular at football matches, has escalated during the last few years, resulting in increasing societal and public health costs and the tragic deaths of two people [3, 4, 13].

Despite alarming reports in international and Swedish media on serious alcohol-related problems at sporting events and demands for action to reduce such problems, knowledge on the general public’s opinion of these disturbances and its opinions towards possible preventive interventions is limited.

International literature suggests that the general support for community-based alcohol-control policies is strong [14–18]. However, public opinion might vary between countries and within subgroups of a population. Typically, people tend to be more positive towards a policy that does not affect their own lifestyle. For example, in a large survey of opinions towards alcohol-control policies in the adult U.S. population, the highest level of support for alcohol-reducing regulations was given by women, older adults (>25 years), parents, and infrequent or non-drinkers of alcoholic beverages [15]. Moreover, a Swedish population survey on public opinion regarding alcohol service at licensed premises showed an overall strong support for the practice of responsible beverage service and for stricter enforcement of existing legislation. Importantly, however, the level of support was influenced by measured background factors showing that frequent alcohol consumers, men, young people (<30 years), and frequent visitors to licensed premises were less supportive of interventions and restrictions [19]. Public opinion and support for alcohol policies are important and can influence the decisions of policymakers and facilitate the implementation of policy changes [15]. Increased understanding of the public opinions and community readiness towards alcohol sales, consumption and intoxication at sporting events can help tailor and implement future community-based interventions. Furthermore, interventions to reduce adverse consequences of alcohol intoxication at sporting events have previously been shown to decrease violence and public health costs and could consequently increase the positive contribution that football offers to the community [20, 21].

Football is the largest sport globally and the most popular sport in Sweden, attracting the greatest number of spectators and supporters. For instance, the three largest cities of Sweden (Stockholm, Gothenburg and Malmö) have sporting arenas hosting Swedish Premier Football League (SPFL) matches, with the capacity for between 18,000 and 50,000 spectators. The arenas are located in the central part of, or just outside, the city centres. There are a number of licensed premises in the close vicinity of all arenas at which alcohol can be purchased. At the arenas, there are kiosks and booths serving medium strength beer (3.5% by volume) and bars and pubs serving all types of alcohol, including full strength beer (>4.5% by volume) and hard liquor. SPFL matches are scheduled on weekday evenings or during daytime on weekends in order to reduce alcohol-related problems. At high-risk matches, such as local derbies, alcohol sales at the arenas are limited to medium strength beer. Security guards, uniformed and civilian police officers and licensed security staff circulate inside and within the arena and in close reach of arena entrances.

In 1996, our research unit STAD (an abbreviation for “Stockholm Prevents Alcohol and Drug Problems”) was instated by the Stockholm County Council with the aim to reduce problems related to heavy alcohol use, intoxication, and violence at licensed premises through community-level methods. Interventions developed by STAD have shown to be effective in reducing the serving of alcohol to under-aged [22] and obviously intoxicated persons [23], the frequency and cost of alcohol-related violent crimes [24–26], and emergency room visits [27]. In 2015 STAD initiated a novel project aiming to reduce problems related to alcohol intoxication among spectators at sporting events in Sweden [28]. The current study is a part of this project with the overall aim to explore public opinions on alcohol use, intoxication and alcohol policies at professional football matches, taking demographic background factors and individual characteristics into consideration. The key research questions were:

1. To what extent does public opinion support alcohol policies, prevention strategies and sanctions to reduce alcohol intoxication at football matches?
2. To what extent does public opinion perceive that alcohol intoxication constitutes a problem at football matches?
3. Do behavioural or individual characteristics influence public opinion towards alcohol intoxication, policies, prevention strategies and sanctions to reduce alcohol consumption at football matches?

## Methods

### Sample and survey

Data was collected using web-based interviews with individuals included in a web panel (”Novus Sverigepanel”) [29] during the period February 5^th^ to 19^th^ 2016. The study time was selected to reflect a period outside the ongoing series to avoid bias due to media coverage on alcohol-related disturbances at professional football matches. The panel abides by the international ICC/ESOMAR standards on market and social research [30], and comprises approximately 40,000 individuals randomly recruited through national telephone surveys. The sample is selected to reflect a population in the age range of 18–79 years, representative in regards to age, sex and geographic distribution. To avoid skewedness of the panel, personal invitations are sent to underrepresented groups. Potential biases in the panel structure are remedied by a nationally representative sample drawn from the panel and data is weighted using a post-stratification procedure to correct for differences between the achieved sample and the Swedish population with respect to gender, age, region of residence and political affiliation. All participants included in Novus’ web panel receive a fixed annual reimbursement for participation. No specific or additional incentive was dispensed for participation in the current study. All reference values in each post-stratum were established based on 2010 Statistics Sweden census data [31].

A total of 5225 individuals aged 18–79 years were drawn based on a random number list and received an invitation to the survey via email. The email contained a brief description of the study and a link directing the respondent to the questionnaire. Individuals who had not responded within the given time period or as a result of a follow-up email invitation were defined as non-responders. A total of 3503 individuals participated in the survey resulting in a response rate of 68%. More specifically, 1083 interviews were performed with individuals representative of the general public and 2420 interviews with individuals selected from urban or suburban areas (i.e., the counties with the three largest cities in Sweden: Stockholm (*n* = 1008), Gothenburg (*n* = 811), and Malmö (*n* = 679)). These counties were chosen to reflect areas hosting the majority of the SPFL events in Sweden.

### Measures and definitions

#### The questionnaire

The questionnaire contained 36 questions covering the following four domains.

The first domain, which included questions 1 to 9, described the demographic distribution of the sample. The second domain (questions 10 to 16) measured individual interest in football and motives for attending or not attending football matches. Individuals who had visited at least one match at a sporting arena during the last season (autumn 2015) were defined as frequent arena visitors. The third domain (questions 17 to 29) assessed opinions on alcohol use at football events and opinions on strategies for reducing intoxication among fellow arena visitors using a five-grade scale. The fourth domain (questions 30 to 36) explored the respondents’ own alcohol use patterns. To assess hazardous alcohol use, the Alcohol Use Disorders Identification Test - Consumption questionnaire (AUDIT-C) was applied. It consists of the first three items of the original 10-item AUDIT screening instrument, which reliably identifies hazardous alcohol use. Each question in the AUDIT-C is answered on a five-point Likert scale and the total score ranges from 0 to 12 [32]. Cut-off scores of ≥ 4 for women and ≥ 5 for men were used to define hazardous alcohol use, derived from the definition established by the Swedish Health and Human Services Department’s national guidelines for disease prevention methods [33].

### Statistical analyses

Descriptive statistics (i.e., means, standard deviations, frequencies and ranges) were used to describe the sample. Apart from descriptive analyses, Pearson’s *χ*2 statistics (2-tailed) were used to investigate differences in proportions, while t-tests for independent samples were used to explore differences between means.

The five-point scale assessing support for the different statements was dichotomized, where 4 and 5 were chosen to indicate support for the statement, and 1–3 were non-supportive. Demographic variables were either dichotomized (i.e., gender, frequency of attendance to arena football events, AUDIT-C scores for hazardous alcohol use and level of educational) or treated as continuous variables (i.e., age) and fitted into eleven multiple logistic regression models estimating their impact on opinions towards alcohol consumption and strategies to reduce intoxicated behaviour at football events. In all regression models, the independent variables were entered simultaneously, given that we had no hypotheses about the relative importance of each variable [34]. The Odds Ratio (OR) for supporting a) statements that alcohol consumption and intoxication are perceived as problematic, and b) strategies to reduce adverse outcomes associated with alcohol consumption and intoxication at sporting arenas were analysed. Data were analysed using SPSS Statistics 20 (IBM Corporation) and results were based on weighted data [35]. For all analyses, a significance level of *p* < 0.05 was used.

## Results

### Demographic distribution of the sample

Table 1 shows the distribution of demographic variables for the overall sample, as well as for the sample representative of the general public and the sample of individuals residing in urban areas, respectively. There was an equal distribution of all the demographic variables analysed (i.e., gender, cohabiting with children, age, marital status, highest education level, and AUDIT-C scores) across the general public and the urban samples, and these were subsequently merged and treated as an overall sample. An average AUDIT-C score of 3.10 was found for the overall sample and approximately 29% of the respondents met the criteria for hazardous alcohol use (AUDIT-C score of ≥ 4 for women and ≥ 5 for men). A larger proportion of male respondents (31%) fulfilled the criteria for hazardous alcohol use compared with female respondents (27%) (*χ*2 (1, 3478) = 6.972, *p* = 0.008). Nearly 12% of the overall sample reported being frequent visitors to arena football events, i.e., having visited either the SPFL or the second highest division (Superettan) on at least one occasion during the past football season. Moreover, frequent visitors to the arena (*M* = 3.80) had a statistically significantly higher total score on the AUDIT-C compared with non-frequent visitors (*M* = 2.98) (t (3455) = 7.964, *p* < 0.001).

**Table 1:** Participant characteristics for the overall sample, the general public sample, and the urban sample. Means (*M*), standard deviations (*SD*), frequencies, ranges and *p* values for comparative statistics shown

Variables	Overall sample (*n* = 3503)	General public sample (*n* = 1083)	Urban sample (*n* = 2420)	*p* ^a^
Gender, % (*n*)				NS
Men	49.9 (1747)	50.3 (544)	49.7 (1203)	
Women	49.4 (1731)	49.4 (535)	49.5 (1197)	
Other	0.7 (24)	0.4 (4)	0.8 (20)	
Mean age, *M* (*SD*, range)	46.6 (16.3, 18–79)	46.7 (16.4, 19–79)	46.5 (16.2, 18–79)	NS
Marital status^b^, % (*n*)				NS
Married/In registered partnership	43.9 (1538)	43.7 (473)	44.0 (1065)	
Other^c^	32.0 (1119)	31.3 (339)	32.3 (781)	
Cohabiting with children, % (*n*)	33.9 (1186)	34.5 (374)	33.6 (812)	NS
Highest level of education, % (*n*)				NS
Elementary school	6.5 (228)	7.2 (78)	6.2 (150)	
Senior high school	42.4 (1485)	44.6 (483)	41.4 (1003)	
Folk high school	2.5 (89)	2.7 (30)	2.4 (59)	
University/college	48.5 (1701)	45.5 (493)	49.9 (1208)	
AUDIT-C scores				
Mean AUDIT-C total score, *M* (*SD*, range)	3.10 (2.00, 0–12)	3.07 (1.96, 0–11)	3.11 (2.01, 0–12)	NS
Men and women with hazardous alcohol use^d^, % (*n*)	28.5 (998)	27.9 (302)	28.8 (696)	NS
Men with hazardous alcohol use, % (*n*)	30.7 (536)	30.1 (164)	31.0 (373)	NS
Women with hazardous alcohol use, % (*n*)	26.6 (461)	25.8 (138)	27.0 (323)	NS
Type of visitors to football arenas, % (*n*)				NS
Frequent visitors^e^	11.6 (406)	10.8 (117)	11.9 (289)	
Non-frequent visitors^f^	87.1 (3053)	88.5 (959)	86.5 (2094)	
Reported “Don’t know”	1.3 (45)	0.7 (8)	1.5 (37)	

^a^Comparisons between the general public and the urban samples. NS: non-significant *p* values (i.e., ≥ 0.05)

^b^Data missing for seven participants

^c^E.g., single, widowed or divorced

^d^Hazardous alcohol use defined as an AUDIT-C score of ≥ 4 for women and ≥ 5 for men

^e^Frequent visitors were defined as participants who reported having attended a football match in the Swedish Premier Football League (SPFL) or the second highest division on one or more occasions during the past football season (i.e., the football season 2015)

^f^Non-frequent visitors were defined as participants who reported not having attended a football match in the SPFL or the second highest division during the past football season (i.e., the football season 2015)

A total of 688 respondents (19.6%) in the overall sample reported having chosen not to attend a public football match for reasons directly associated with the event. The main reasons for choosing not to attend a football event were disturbances and turbulence directly related to the matches (44.2%) and alcohol-related disturbances due to the intoxication of others (26.7%). Furthermore, among those who might bring children to football matches (*n* = 1186), 220 individuals (18.5%) had chosen not to do so. Among these, 109 individuals (49.6%) reported reasons directly related to the alcohol intoxication of other arena visitors.

### Opinions on alcohol use at football events and opinions on strategies for reducing alcohol intoxication

Table 2 shows the proportion of respondents in the overall sample and among frequent and non-frequent visitors to football arenas who agreed with the statements made concerning alcohol intoxication at football arenas (i.e., questionnaire items 17–29).

**Table 2:** The proportion of respondents who agree with various statements concerning alcohol and intoxication at football arenas. Proportions are shown for the overall sample and among frequent^a^ and non-frequent visitors^b^ to football arenas

	Overall sample	Frequent visitors to football arenas	Non-frequent visitors to football arenas	*p* ^c^
Statement^d^	% (*n*)	% (*n*)	% (*n*)	
Alcoholics beverages should be sold at football arenas	26.1 (827)	46.1 (183)	23.2 (644)	<0.001
Medium strength beer should be sold at football arenas	42.9 (1357)	62.8 (247)	40.1 (1110)	<0.001
Full strength beer/wine should be sold at football arenas	23.3 (746)	40.6 (161)	20.9 (585)	<0.001
Hard liquor should be sold at football arenas	8.7 (281)	13.6 (54)	8.0 (227)	<0.001
Obviously intoxicated persons should be denied entrance to football arenas	93.1 (3047)	92.3 (373)	93.2 (2674)	NS
Obviously intoxicated spectators should be denied alcohol service at football arenas	94.7 (3093)	95.3 (385)	94.6 (2708)	NS
Obviously intoxicated spectators should be evicted from the arena	91.8 (2980)	89.6 (354)	92.1 (2626)	NS
There should be sections at football arenas where alcohol cannot be purchased	65.1 (1912)	50.0 (189)	67.4 (1723)	<0.001
The number of bars and booths selling alcohol at football arenas should be reduced	64.2 (1933)	44.8 (174)	67.1 (1759)	<0.001
More people would attend football matches if the number of obviously intoxicated spectators was reduced	71.0 (1994)	47.2 (175)	74.6 (1819)	<0.001
The atmosphere at the arena grandstand would improve if the number of obviously intoxicated spectators was reduced	77.5 (2276)	49.9 (194)	81.7 (2082)	<0.001

^a^Frequent visitors were defined as participants who reported having attended a football match in the Swedish Premier Football League (SPFL) or the second highest division on one or more occasions during the past football season (i.e., the football season 2015)

^b^Non-frequent visitors were defined as participants who reported not having attended a football match in the SPFL or second highest division during the past football season (i.e., the football season 2015). A total of 45 individuals who responded, “don’t know” were excluded from the analyses

^c^Comparisons between frequent and non-frequent visitors. NS: non-significant *p* values (i.e., ≥ 0.05)

^d^Respondents who responded “no opinion” to a statement were excluded from that analysis. The analyses comprised between 2808 and 3274 individuals, respectively

A minority of the overall sample (26%) supported the sale of alcohol at Swedish football arenas. However, there was a significantly higher support for allowing alcohol sales among frequent visitors to football matches (46%), as compared with non-frequent visitors. The degree of support for selling alcohol at football arenas decreased in all three groups along with increasing alcohol content of beverages. Among the overall sample, 43% supported the sale of medium strength beer (3.5%), 23% supported the sale of full strength beer (>4.5%) or wine and 9% supported the sale of hard liquor at football events.

Furthermore, the results show a strong consensus for preventive actions to reduce the number of obviously intoxicated persons at public football events. Interventions, such as denying entrance and/or evicting intoxicated spectators from the arena, were supported by at least 92% of the respondents in the overall sample.

A majority (71%) believed that attendance at football matches would increase, and 78% thought that the overall atmosphere at the grandstands would improve, if the number of intoxicated individuals was decreased. The support for interventions directly targeting severely intoxicated spectators, (i.e., denying intoxicated spectators entrance to the arenas, alcohol service at the arenas (93 and 95%), and evicting intoxicated spectators from the arenas (92%)) was significantly higher than support for indirect interventions (i.e. assigning sections at football arenas where alcohol cannot be purchased (65%) and reducing the number of bars and booths selling alcohol at the arenas (64%)), (all Chi-squares test *p* < .0001).

### Factors associated with opinions of alcohol use and intoxication at football arenas

Table 3 shows the results of the multiple logistic regressions and how support for the various statements in the questionnaire concerning alcohol and intoxication at football arenas were associated with gender, age, level of education, arena football event attendance and alcohol use. Overall, male gender was associated with several of the outcomes associated with opinions towards alcohol use at football events and potential interventions and restrictions to reduce alcohol intoxication, whereas age was of only marginal importance for the same outcomes. More specifically, the odds of support for selling alcohol at professional football events among men relative to women were 3.5 to 1. Consequently, male gender corresponded with non-support for restrictions in alcohol-selling policies (OR_AlcFreeSections_ = 0.8 and OR_RestrictBars_ = 0.4) and low belief in the effect of actions to reduce alcohol intoxication on the attendance rate and overall atmosphere at professional football events (OR_Attendance_ = 0.6 and OR_Atmosphere_ = 0.4).

**Table 3:** Results of logistic regressions predicting support for various statements concerning alcohol and intoxication at football arenas^a^

Statement (dependent variable)	Male gender OR (95% CI), *p* ^b^	High level^c^ of education OR (95% CI), *p*	Frequent visitor at a football arena^d^ OR (95% CI), *p*	Hazardous alcohol use^e^ OR (95% CI), *p*
Alcoholics beverages should be sold at football arenas	3.5 (2.9–4.3), <0.001	NS	1.8 (1.5–2.3), < 0.001	2.4 (2.0–2.9), < 0.001
Medium strength beer should be sold at football arenas	2.7 (2.3–3.2), <0.001	NS	1.7 (1.3–2.1), < 0.001	2.1 (1.8–2.5), < 0.001
Full strength beer/wine should be sold at football arenas	4.1 (3.4–5.0), <0.001	NS	1.6 (1.3–2.1), < 0.001	2.7 (2.2–3.2), < 0.001
Hard liquor should be sold at football arenas	3.6 (2.7–4.8), <0.001	NS	NS	1.9 (1.5–2.5), < 0.001
Obviously intoxicated persons should be denied entrance to football arenas	NS	NS	NS	0.7 (0.5–0.9), 0.018
Obviously intoxicated spectators should be denied alcohol service at football arenas	NS	NS	NS	NS
Obviously intoxicated spectators should be evicted from the arena	NS	NS	NS	NS
There should be sections at football arenas where alcohol cannot be purchased	NS	NS	0.5 (0.4–0.6), < 0.001	NS
The number of bars and booths selling alcohol at football arenas should be limited	0.4 (0.4–0.5), <0.001	NS	0.6 (0.5–0.7), < 0.001	0.6 (0.5–0.7), < 0.001
More people would attend football matches if the number of obviously intoxicated spectators was reduced	0.6 (0.5–0.7), <0.001	NS	0.4 (0.3–0.5), < 0.001	0.7 (0.6–0.9), 0.001
The atmosphere at the arena grandstand would improve if the number of obviously intoxicated spectators was reduced	0.4 (0.3–0.5), <0.001	1.3 (1.1–1.6) 0.010	0.3 (0.2–0.4), < 0.001	0.6 (0.5–0.8), < 0.001

^a^All models were adjusted for age

^b^Odds Ratios (ORs), 95% Confidence Intervals (CIs) and *p* values presented. NS: non-significant *p* values (i.e., ≥ 0.05)

^c^University/college/folk high school vs elementary school/senior high school

^d^Frequent visitors were defined as participants who reported having attended a football match in the Swedish Premier Football League (SPFL) or the second highest division on one or more occasions during the past football season (i.e., the football season 2015)

^e^Hazardous alcohol use defined as an AUDIT-C score of ≥ 4 for women and ≥ 5 for men

Being a frequent visitor at football arenas corresponded to a supportive opinion towards alcohol-containing beverages at football events (OR_Alcohol_ = 1.8). Furthermore, frequent arena visitors were non-supportive of interventions aiming to restrict alcohol availability at arenas (OR_AlcFreeSections_ = 0.5 and OR_RestrictBars_ = 0.6) and showed low belief in the effect of alcohol-restricting actions or interventions on the attendance at football events, or the atmosphere at the arenas (OR_Attendance_ = 0.4 and OR_Atmosphere_ = 0.3).

Individual hazardous alcohol use corresponded to the level of support for selling alcohol-containing beverages at football arenas (OR_Alcohol_ = 2.4), including support for selling hard liquor (OR_HardLiquor_ = 1.9). Furthermore, hazardous alcohol use was associated with non-support for interventions targeting alcohol intoxication in arena spectators, such as denying entrance (OR_Entrance_ = 0.7) and reducing the number of bars and booths selling alcoholic beverages at football arenas (OR_RestrictBars_ = 0.6).

## Discussion

In this cross-sectional web survey, we assessed the public opinion towards alcohol use, intoxication and alcohol policies at professional football matches in Sweden. We found a strong consensus for stricter alcohol policies and sanctions at football matches.

Firstly, the current study shows that the support for alcohol sales at sporting events is surprisingly low (26%) and that there is strong public support for policies and actions to reduce intoxication at the arenas (Table 2). Overall over 90% of the respondents suggested obviously intoxicated patrons should be denied entrance, denied further alcohol service, and evicted from the arena. These findings are in accordance with previous studies showing strong support for alcohol-control policies in “high-risk” environments such as licensed premises (i.e., bars and clubs) [14–19, 22–27].

Secondly, our results show that the public does perceive disturbances and incidents related to alcohol intoxication at professional football matches to be a substantial problem. These disturbances significantly and directly affect the respondents’ motivation to attend professional football matches at public arenas. Almost one in every five respondents reported opting out of attending a professional football match due to reasons directly associated with the match, such as alcohol intoxication among other spectators. This is in agreement with reports in Swedish media on fear of increasing violence associated with SPFL matches [3, 4] as well as mobilization among Swedish policymakers, authorities and key stakeholders to reduce disturbances at and near Swedish football arenas. Importantly, a proportionally large percentage of those who chose not to bring children to football matches reported reasons related to alcohol intoxication among other arena visitors. This becomes a particularly serious setback for the overall positive contribution of football to the youth and adolescent community. Thirdly, an important aim of this study was to explore whether opinions towards alcohol at sporting events were influenced by individual background factors and/or behavioural characteristics. Despite an overall agreement for regulations on alcohol serving practices and intoxication at football matches, our results show some important differences in public opinion across demographics and behavioural subgroups. Male gender was significantly associated with a supportive opinion towards alcohol consumption at football events and a non-supportive opinion towards community-based interventions to control alcohol consumption and reduce the negative influence of intoxicated spectators. In general, frequent arena visitors and hazardous drinkers report a less restrictive opinion towards alcohol use, intoxication and alcohol policies relative non-frequent visitors and non-hazardous drinkers (Table 3). These observations are in accordance with previous studies showing that non-drinkers or moderate consumers of alcohol tend to be more supportive of alcohol-control policies compared with those who might be most affected by the regulations [15–17, 19, 36]. Furthermore, our results are in concordance with literature, showing that individuals overall tend to be more inclined to support interventions and restrictions that do not directly influence their own choices or behaviours.

The strong public support for community-based interventions to reduce intoxication levels at football events shown in our study will facilitate the implementation of intervention strategies. Previous research by Drygas et al. [37] suggests that public arenas have considerable potential to become settings for health promoting interventions.

The results need to be interpreted in light of some limitations. First, this is a cross-sectional study based on a web survey. The results must therefore primarily be viewed as representative for the given time point and the sample is not, by strict definition, a random sample from the general population, but rather randomly selected from a panel. Furthermore, some of the prior studies exploring public opinion on intoxicated behaviour were performed in countries and cultures that may differ from the Swedish setting. Several factors such as age limits, alcohol sales, opening hours, alcohol marketing and promotion, alcohol prizing and traffic laws may potentially influence alcohol culture and opinion in different countries. Generalization across time periods and to different populations should consequently be done with caution. Importantly, however, the panel abides to the international ICC/ESOMAR standards on market and social research and includes randomly recruited individuals. Secondly, the question of selective non-response needs to be addressed. However, while other researchers have raised issues on selective non-response in surveys on alcohol consumption, the magnitude of any such potential problem is, to our knowledge, still unknown [38] and might partly be accounted for by employing the weight procedure applied in the current study. Thirdly, the level of support given to any particular statement might depend on how the questions are worded [15, 19]. The questionnaire in the present study was developed uniquely to fit the design of the present study and has consequently not yet been validated in this or any other population. It therefore becomes difficult to estimate if and how the results would have been affected, had the questions been phrased differently. Although it is difficult to appreciate the impact on the internal validity of the results, the novelty of this research area leaves no validated alternatives to questionnaires, as far as we know.

Our study has a number of key strengths, including a randomly recruited sample from a panel comprising approximately 40 000 individuals randomly recruited from the entire Swedish population. The sample was weighted using a post-stratification procedure to correct for potential differences between the achieved sample and the source population. Moreover, a web-based design guarantees participants anonymity and minimizes the risk for interviewer bias and bias due to fear of negative consequences related to certain answers. Furthermore, public opinion surveys typically suffer from low response rates [39]. A response rate of 68% for the current study increases both the validity and the generalizability of our findings. Also, the panellists used were restricted to 12 studies per year, to avoid recruiting pundits answering surveys for other than the intended reasons.

An important reflection in light of the present findings of low support for alcohol sales at professional football matches is the fact that alcohol consumption is considered to be a focal point of many sporting events. Previous research shows that adult and, in particular, adolescent alcohol drinking behaviours are influenced by marketing and advertising promoting alcohol consumption [40, 41]. The alcohol industry specifically targets sporting events with advertisements, commercials for alcohol-containing beverages, and sponsorship, while little is known about how this commercial association between alcohol consumption and sporting events may influence children and young people [42].

## Conclusions

Given the well-established disinhibiting effects of alcohol [43], it might be reasonable to hypothesize that the overall levels of disturbances and turbulence at or near football arenas could be reduced through decreased alcohol consumption [9, 11]. Further knowledge regarding opinions towards alcohol-related disturbances and violence is crucial in order to tailor the most efficient and appropriate interventions. Previous studies show the importance of exploring public opinions and community readiness, suggesting that local public opinion can be a trigger for policy development [44]. Moreover, community-based interventions including strategies such as community mobilization and media advocacy can be used to change public opinion on alcohol policies [45].

Overall, our results indicate a substantial interest for professional football in Sweden. Given reports that the overall atmosphere surrounding live matches is an important incentive to attend arena football, disturbances directly or indirectly related to alcohol consumption remain important to understand and resolve [46]. The importance of exploring public perception of alcohol intoxication among spectators at football matches is further highlighted by the fact that a substantial portion of the respondents report actively abstaining from attending and bringing children to football events due to disturbances directly related to the matches.

Based on our previous experiences of community-based interventions to reduce alcohol intoxication and illicit drug use in night-life settings, we have initiated a similar approach at football events. Strategies used in this novel approach include community mobilization, training, policy work, increased enforcement, and media and PR work. The current study provides further understanding of the opinions on alcohol use, and intoxication in relation to professional football events, and shows strong public support for community-based interventions to reduce intoxication levels at these events.
